# Scleral lens for severe dry eye status post lacrimal gland resection for adenoid cystic carcinoma

**DOI:** 10.1016/j.ajoc.2020.100601

**Published:** 2020-01-24

**Authors:** Daniel J. Oh, Raman Michael, Pete Setabutr, Ellen Shorter

**Affiliations:** Department of Ophthalmology and Visual Sciences, University of Illinois at Chicago, Chicago, IL, USA

**Keywords:** Scleral contact lens, Adenoid cystic carcinoma

## Abstract

**Purpose:**

Scleral contact lenses (SCLs) are devices that allow a fluid reservoir between the contact lens and the cornea, providing both improved lubrication and refraction. Consequently, SCLs have been used for significant refractive error in addition to a wide range of ocular surface diseases. We present the first case of a woman who complained of severe dryness and pain following resection of an adenoid cystic carcinoma of her lacrimal gland with complete resolution of her symptoms with a SCL.

**Observations:**

A woman who complained of severe dryness and pain following resection of an adenoid cystic carcinoma of her lacrimal gland presented to the ophthalmology clinic. She had no subsequent lacrimal function without relief from conventional dry eye treatments. However, early treatment with a SCL successfully preserved her ocular surface, improved her corneal staining pattern, and improved her vision.

**Conclusions and Importance:**

While other options exist, including permanent tarsorrhaphy, lid taping, or moisture goggles, the SCL allowed the combination of cosmesis, visual function, and ocular surface rehabilitation.

## 1 Introduction

Scleral contact lenses (SCLs) are rigid devices that are supported by the sclera, completely vaulting over the cornea beyond the corneal limbus. Their earliest uses included correction of corneal refractive errors including astigmatism.^1^ However, with poor oxygen permeability of early designs as well as the advent of corneal and soft contact lenses, SCLs fell out of favor. They began regaining popularity as new technology and lens materials increased their gas-permeability and a variety of ocular surface diseases that could not be treated by soft contact lenses showed benefit from SCLs.^2^ SCLs allow for the maintenance of a fluid reservoir between their posterior surface and the anterior surface of the cornea, providing both improved lubrication and refraction.^3^ In addition to correcting corneal astigmatism, SCLs have successfully treated a variety of diseases of the ocular surface including keratoconus and ectatic disorders, severe astigmatism, Stevens-Johnson syndrome, microphthalmia, and severe dry, especially after soft lenses and punctal occlusion have failed.^3^

The literature has seen numerous publications related to SCLs and their applications increasing over the past three decades. We present a case of a woman whose dry eye and blurry vision status post lacrimal gland resection for adenoid cystic carcinoma (ACC) in which early intervention with SCL has successfully preserved her ocular surface and improved vision.

## 2 Case presentation

A 53-year-old Asian female was referred to the contact lens service by her oculoplastics surgeon for evaluation of severe dry eye in the left eye. She had a prior ocular history of LASIK surgery in both eyes and recent diagnosis of adenoid cystic carcinoma (ACC) of the lacrimal gland. A few months prior to presentation, she noted a droopy left upper eyelid and a painless hard lump in the left superior temporal region. She underwent computed tomography (CT) and magnetic resonance imaging (MRI) of the orbits demonstrating a lesion of the left lacrimal gland. Subsequent biopsy revealed ACC of the lacrimal gland. She underwent orbitotomy with total resection of the lacrimal gland and surrounding tissues (Fig. 1a, Fig. 1b). Her immediate post-operative course was uncomplicated and bi-weekly radiation therapy with proton beam radiation (range 50–65 Cobalt Gray Equivalent) was initiated for eight weeks.

**Fig. 1a:**
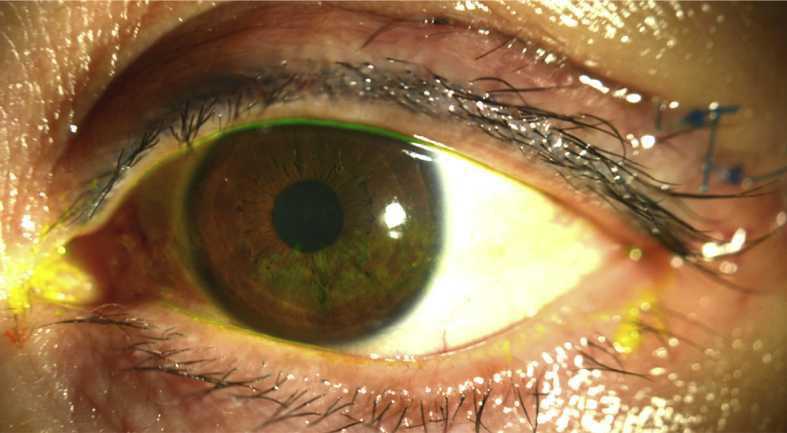
Slit lamp photograph of the left eye at baseline.

**Fig. 1b:**
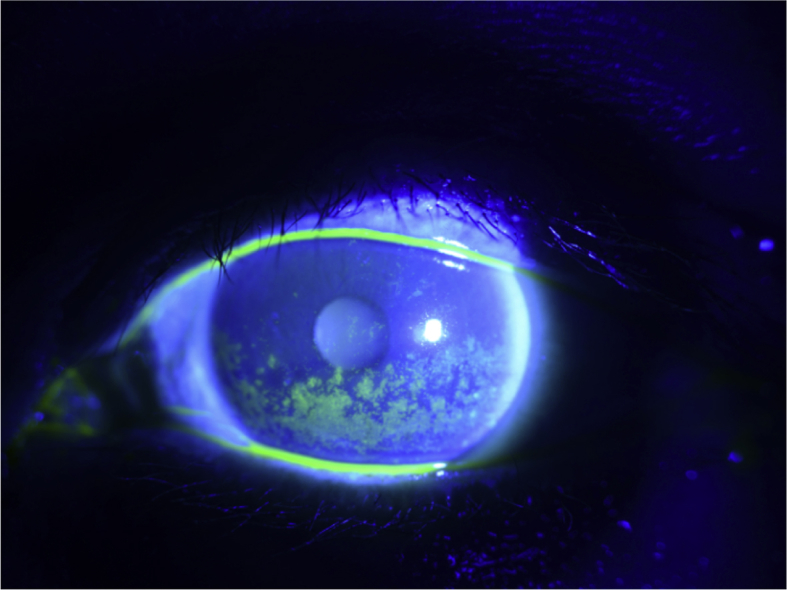
Baseline slit lamp photograph of the left eye with severe diffuse punctate epithelial erosions with fluorescein staining prior to SCL fitting.

At the time of her initial contact lens evaluation, she complained of extreme dryness, mucus discharge and blurry vision in her left eye. Her entering uncorrected vision was 20/20 in the right eye and 20/200 in the left eye improving to 20/50 in the left eye with manifest refraction. Ocular medications included bacitracin ophthalmic ointment four times a day on her eyelid sutures, preservative free artificial tears eight times a day and preservative free lubricating ophthalmic ointment four times a day in the left eye. In addition, in between application of drops and ointment, she was taping her eyelids closed and using a moisture chamber at all times. Pupils were round and reactive without afferent pupillary defect and extraocular motilities were full. Her intraocular pressures were normal in both eyes. The slit lamp examination was notable for mild ptosis of the left eye with 1mm lagophthalmos. She had mild diffuse conjunctival injection and severe diffuse punctate epithelial erosions in the left eye (Fig. 1a, Fig. 1ba and b) while the right eye was unremarkable for eyelid, conjunctival, corneal or lenticular abnormalities. Pachymetry was 532 in the right eye, 523 in the left eye with keratometry of 42.94/41.57 with central flattening in the right eye and 42.50/40.55 with central flattening and surface irregularity in the left eye. Without use of artificial tears, she had a Schirmer's of 0mm in both eyes.

Therapeutic options were discussed including frequent lubrication, moisture chamber use, lid taping and tarsorrhaphy. We proceeded with a trial fitting with an 18 mm gas permeable scleral lens (BostonSight Scleral, Needham, MA) in her left eye (Fig. 2a, Fig. 2b, Fig. 2cA–C). The standard trial had minimal central corneal clearance, mild conjunctival vascular compression temporally and inferiorly however improved vision to 20/20 with astigmatic over refraction.

At the one week follow up visit, the customized 18mm scleral device was dispensed for the left eye (Fig. 2a, Fig. 2b, Fig. 2ca, b, 2c). She returned for evaluation after 3 hours of lens use in the left eye. The vison in the right eye remained stable and the left eye improved to 20/25. The device had adequate central corneal clearance and limbal clearance 360. There was no mid haptic vascular compression or lens edge impingement. After the device was removed, there was very mild punctate epithelial staining but no conjunctival impression or staining of the left eye. She had persistent 1mm lagophthalmos but the erosions were markedly reduced. She was advised to continue daily use of the SCL throughout all waking hours except during her radiation treatment, along with overnight lubricating ophthalmic ointment and moisture chamber goggles.

**Fig. 2a:**
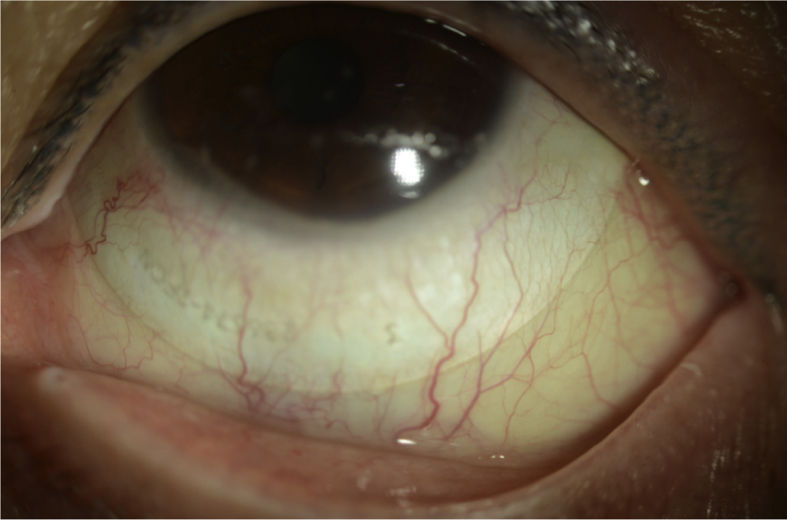
Slit lamp photograph of the SCL fitting in upgaze.

**Fig. 2b:**
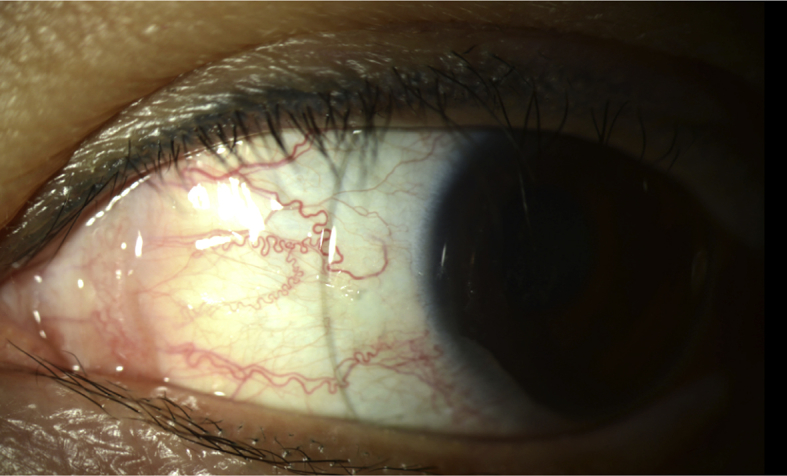
Slit lamp photograph of the SCL fitting in left gaze.

**Fig. 2c:**
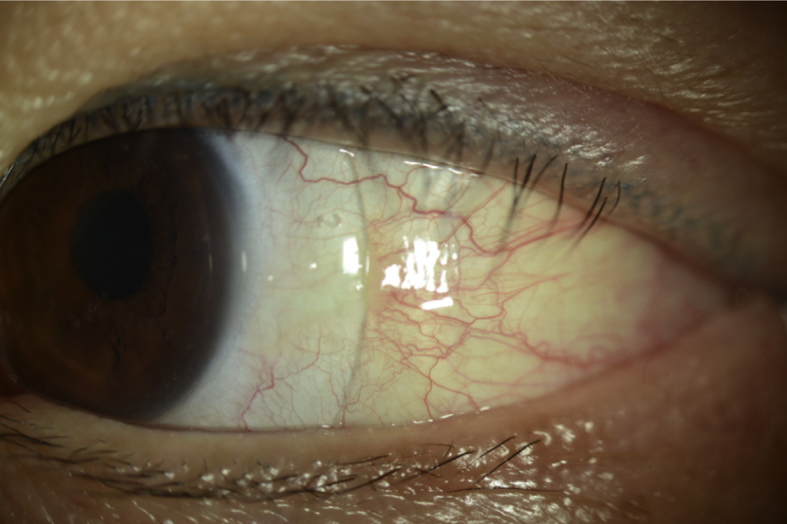
Slit lamp photograph of the SCL fitting in right gaze.

During her 3 month and 1 year follow up visits, the patient reported successful completion of her radiation therapy and daily use of her SCL. She reported improved comfort with daily use of her SCL and frequent preservative free artificial tear use. Despite using artificial tears at least ten times per day, she reported blurred vision worsening later in the day. Her vision remained stable 20/20 in the right eye and 20/20 in the left eye immediately after lens cleaning and reapplication and with everyday use requiring artificial tears daily or twice a daily. While the slit lamp exam and scleral lens fit remained stable, the front surface of the lens demonstrated non-wetting. Devices were ordered with Tangible Hydra-PEG (Contamac) with the goal of improving lens surface wettability. The Tangible Hydra-PEG was successful with resolution of the anterior lens surface.

## 3 Discussion

ACC is a very rare condition that is a variant of adenocarcinoma that has been reported in the salivary gland, skin, breast, lung, prostate, gastrointestinal tract, female genital tract, and lacrimal gland. When arising in the latter, as in our patient, it is often aggressive, and metastases are not uncommon.^4^ Treatment includes a combination of complete tumor excision with post-operative external beam radiation therapy (EBRT). Lacrimal ACC not amenable to complete excision should be removed via exenteration.^4^ With such aggressive treatment and ultimate removal of the lacrimal gland, an inevitable long-term complication in these patients is dry eye, which can often be severe. Individuals should also be monitored for ocular complications due to radiation and steroid use such as radiation keratopathy and cataract formation.

Dry eye syndrome (DES), or keratoconjunctivitis sicca, is a complex disease with numerous etiologies, including resection of the lacrimal gland as in our patient. While traditional therapies begin with conservative artificial tears and lubricating ointments, our patient had no tear production (0 mm on Schirmer testing) and thus required a SCL in addition to copious artificial tears. Thulasi and Djalilian^5^ outline other modalities including anti-inflammatory regimens, typically with a steroid and later an immunomodulator.^6^ Tetracyclines and macrolides, which exhibit some anti-inflammatory properties, have also been proven effective, as have punctal plugs and light and thermal pulsation therapies. For refractory cases, studies have supported the use of autologous serum tears, umbilical cord serum eye drops, neurotrophin eye drops, cryopreserved amniotic membrane transplantation, and contact lenses, among which SCLs demonstrate higher efficacy than soft lenses.^6^^,^^7^

SCLs have generated much excitement for patients and providers. With improvement in technology and manufacturing, there is continued expansion for their use and improved patient outcomes. Schornack^8^ groups these into three categories: ocular surface disease, visual rehabilitation/corneal irregularity, and correction of refractive error. The first category encompasses conditions such as Stevens-Johnson syndrome, chronic graft-vs-host disease (GVHD), exposure and neurotrophic keratopathy, cicatrizing conjunctivitis, Sjogren syndrome, corneal dystrophy/degeneration. The second group includes primary corneal ectasia, penetrating keratoplasty, refractive surgery, and corneal scarring. The third category consists of aphakia and high ametropia.^8^ One lens in particular, Prosthetic Replacement of the Ocular Surface Ecosystem (PROSE; Boston Foundation for Sight, Needham, MA), has enjoyed much success in treating ocular surface disease including limbal stem cell degeneration, Stevens-Johnson syndrome, exposure keratopathy, and graft versus host disease.^9^

Though promising, SCLs have some limitations. Complications can arise from infection, hypoxia, and difficulties with fitting.^10^ Individuals with exposure or lack of tear production should be closely monitored for issues. Lenses are susceptible to deposits, breaks, and poor surface wetting. Tangible Hydra-PEG (Tangible Science, LLC) is a novel solution to this latter problem. The SCL is treated with a 90 percent polyethylene glycol polymer mixture that covalently bonds to the lens surface.^11^ Moreover, from a logistical perspective, SCLs still have relatively low availability, high cost, low awareness of therapeutic effects among providers, and difficulty of use.^6^

This patient highlights the benefits of early, large diameter therapeutic SCL in a patient with a rare condition and no lacrimal function. While other more permanent options including tarsorrhaphy could offer similar improvement in comfort, a SCL allows visual function as well as good cosmesis. A SCL should be considered when traditional dry eye therapies fail or earlier as highlighted in this case when there is little to no tear production. While a SCL can greatly improve quality of life, close follow up with respected oncology center is also critical in improving survival.

Patient consent: Written consent to publish this case has not been obtained. This report does not contain any personal identifying information.

## Authorship

All authors attest that they meet the current ICMJE criteria for authorship.

## Declaration of competing interest

The following authors have no financial disclosures: DO, RM, PS, ES.
